# Association between Personality Traits and Sleep Quality in Young Korean Women

**DOI:** 10.1371/journal.pone.0129599

**Published:** 2015-06-01

**Authors:** Han-Na Kim, Juhee Cho, Yoosoo Chang, Seungho Ryu, Hocheol Shin, Hyung-Lae Kim

**Affiliations:** 1 Department of Biochemistry, School of Medicine, Ewha Womans University, Seoul, South Korea; 2 Center for Cohort Studies, Total Healthcare Center, Kangbuk Samsung Hospital, School of Medicine, Sungkyunkwan University, Seoul, South Korea; 3 Department of Health Sciences and Technology, SAHIST, Sungkyunkwan University, Seoul, South Korea; 4 Biostatistics and Clinical Epidemiology Center, Research Institute for Future Medicine, Samsung Medical Center, School of Medicine, Sungkyunkwan University, Seoul, South Korea; 5 Department of Health, Behavior and Society and Epidemiology, Johns Hopkins Bloomberg School of Public Health, Baltimore, Maryland, United States of America; 6 Department of Occupational Medicine, Kangbuk Samsung Hospital, School of Medicine, Sungkyunkwan University, Seoul, South Korea; 7 Department of Family Medicine and Health Screening Center, Kangbuk Samsung Hospital, School of Medicine, Sungkyunkwan University, Seoul, South Korea; University of Vienna, AUSTRIA

## Abstract

Personality is a trait that affects behavior and lifestyle, and sleep quality is an important component of a healthy life. We analyzed the association between personality traits and sleep quality in a cross-section of 1,406 young women (from 18 to 40 years of age) who were not reporting clinically meaningful depression symptoms. Surveys were carried out from December 2011 to February 2012, using the Revised NEO Personality Inventory and the Pittsburgh Sleep Quality Index (PSQI). All analyses were adjusted for demographic and behavioral variables. We considered beta weights, structure coefficients, unique effects, and common effects when evaluating the importance of sleep quality predictors in multiple linear regression models. Neuroticism was the most important contributor to PSQI global scores in the multiple regression models. By contrast, despite being strongly correlated with sleep quality, conscientiousness had a near-zero beta weight in linear regression models, because most variance was shared with other personality traits. However, conscientiousness was the most noteworthy predictor of poor sleep quality status (PSQI≥6) in logistic regression models and individuals high in conscientiousness were least likely to have poor sleep quality, which is consistent with an OR of 0.813, with conscientiousness being protective against poor sleep quality. Personality may be a factor in poor sleep quality and should be considered in sleep interventions targeting young women.

## Introduction

Sleep is a universal part of life and one of the important behavioral components of sustaining health state. Sleep disturbance is linked to the development of physical and psychological problems, including cardiovascular disease, diabetes, obesity, anxiety, depression, and all-cause mortality [1–7]. An estimated 50–70 million US adults have a sleep or wakefulness disorder [8], and the prevalence of insomnia in the adult Korean population is greater than 20% [9]. Currently, most assessments of sleep quality depend upon individual perception, although it can be presented as objective sleep parameter through itemizing and measuring. Such perception is likely to depend upon the personality of the individual. Personality has been reported as a predictor of the objective sleep parameters as well [10, 11]. This suggests that the link between personality and sleep may be important in the assessment of sleep quality and in intervention. An increasing number of behavioral therapy intervention trials seek to improve sleep quality by tailoring interventions to individuals’ needs and stages of change [12–14]. Thus, it is important to identify individual-level variables associated with poor sleep quality, particularly personality traits.

The five-factor model (FFM) of personality has emerged as a promising predictor of health behaviors [15]. The five-factor model (FFM) is a comprehensive yet manageable taxonomy of traits that is generalizable across cultures, including that of Korea [16, 17]. According to the FFM, personality traits can be described using five broad dimensions, also known as the Big Five personality domains: Neuroticism (N), Extraversion (E), Openness to Experience (O), Agreeableness (A), and Conscientiousness (C). Each factor is defined by lower-order traits, known as facets. Of these five factors, neuroticism and conscientiousness have been frequently linked to a variety of health-risk behaviors and outcomes [18]. For example, high neuroticism or low conscientiousness was associated with lower subjective [19] and physician-rated health [20], chronic illnesses [21], and mortality [22].

Recently, Duggan reported that low conscientiousness and high neuroticism were the best predictors of poor sleep (i.e., poor sleep hygiene, low sleep quality, and increased sleepiness) [23]. They created new variables using principal component analysis (PCA) to remove multicollinearity between the original personality factor scores. When they re-ran analyses using men-centered personality traits, the conscientiousness parameter was in the same direction but became non-significant, whereas the neuroticism parameter remained significant in the multiple linear models. Hintsanen et al., also reported that the conscientiousness was significantly associated with sleep deficiency when it was examined separately as a predictor, but when all other personality traits were additionally added, the associations attenuated to non-significant [24]. In the presence of correlation among predictors, focusing solely on regression weights yields at best limited information and, in some cases, erroneous interpretation in the multiple regression model [25]. Some researchers have suggested that structure coefficients must be interpreted in conjunction with the standardized beta weights when predictors are correlated [26, 27]. Williams et al. examined the independent and interactive contributions of neuroticism and conscientiousness to sleep quality, but they also interpreted only beta weights in the regression model [28]. Thus, it seems important to understand both the unique and common (i.e. shared) effects of personality traits for the sleep quality.

Several other studies have explored the relationship between sleep quality and neuroticism or conscientiousness, but most of them have been conducted in university students, clinical samples, or small samples [23, 28–31]. Recently, sleep quality has emerged as an important issue in young adult, particularly workers. Doi et al. found that being younger was a risk factor for poor sleep quality, arguing that younger workers may be more vulnerable to stressors because they are less able to cope [32, 33]. In young adults, good sleep quality might make them healthy and sustain productive activity. However, there little research of sizable samples to identify personality traits related with sleep quality in young adults. Thus, there is a need to assess the relationship between sleep quality and personality for effective health-care and preventive health interventions in young adults.

Many studies have reported sex differences in personality traits, and the prevalence of insomnia and depression [34, 35]. Several studies have reported a relationship between personality traits and sleep health in Caucasians [23, 29]. Lichstein et al. have documented racial differences in sleep [36]. Although there are several studies in Asians, most of them have been conducted in adolescents, university students, or small samples [30, 37, 38]. Besides, they focused on only the neuroticism. It needs to be examined whether the sleep quality would be related with the five factors of personality in broad range of age.

Our cross-sectional, survey-based study, which analyzes the five broad dimensions of personality as well as specific facets, was carried out in a sample of young Korean women not reporting clinically depression symptoms. This study was performed in a cohort that based on health screening for middle-aged workers and their spouses. Our aim was to determine whether an individual’s personality traits significantly are associated with her sleep quality in young women.

## Materials and Methods

### Participants

**Fig 1:**
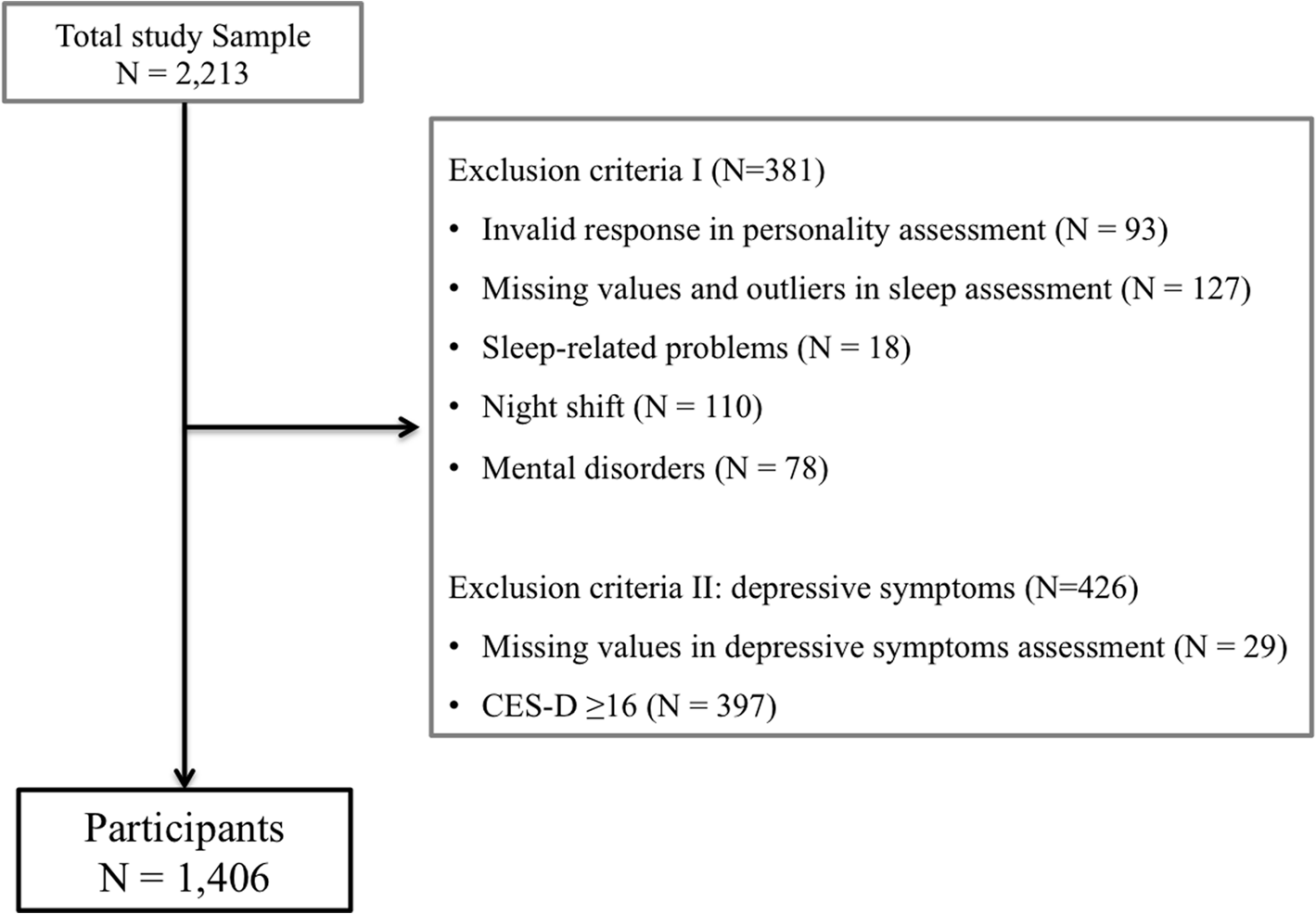
Sample selection procedure. Some participants were excluded for multiple criteria.

Participants were recruited from the Kangbuk Samsung Health Study, which is a cohort study of Korean men and women who undergo a comprehensive annual or biennial examination at Kangbuk Samsung Hospital Screening Centers in South Korea [39]. The sample for this analysis included all 2,213 female participants between the ages of 20 and 40 who underwent a comprehensive health checkup and completed a sleep quality questionnaire. The Revised NEO Personality Inventory (NEO PI-R) manual provides a protocol for validity checking based on acquiescence, naysaying, and randomness of response. Based on item response patterns in the personality assessment, 93 subject surveys were classified as invalid and eliminated from this analysis. These subjects provided repetitive answers or had a pattern of acquiescing or naysaying that would have invalidated formal scoring and interpretation of the NEO PI-R. In addition, 106 subjects with one or more missing values, or 21 subjects who showed the abnormal habitual sleep efficiency according to the sleep assessment were excluded. We also excluded participants with a history of sleep-related problems (N = 18). Additionally, we excluded participants who worked night shifts (N = 110) and those diagnosed with mental disorders such as depression and panic disorder (N = 78). Depression symptoms were measured using the Korean version [40] of the Center for Epidemiologic Studies Depression Scale (CES-D) [41]. Participants who had symptoms of depression (CES-D ≥16) [42] and missing values were excluded from the analysis (N = 426). In the end, the total number of subjects included in the study was 1,406 (Fig 1).

### Statement of ethics

The Institutional Review Board of Kangbuk Samsung Hospital approved this study, and written informed consent was obtained from all participants. All applicable institutional and governmental regulations concerning the ethical use of human volunteers were followed during our research.

### Sleep Variables

Sleep quality was measured using the Korean version of the Pittsburgh Sleep Quality Index (PSQI) [43]. The PSQI is a validated, self-administered questionnaire used to generate seven component scores. It contains 19 items, which measure subjective sleep quality, sleep latency, sleep duration, habitual sleep efficiency, sleep disturbances, use of sleep medication, and daytime function. Each component score is weighed equally on a scale from 0 to 3, which are summed together to yield a global PSQI score. This score can range from 0 to 21, with higher scores (≥6) indicating poor sleep quality.

### Personality Assessment

Personality traits were assessed online using the Korean short version of the original NEO PI-R [44], a 90-item measure of the five factors of personality (PSI Consulting Corp., Seoul, Korea). The Korean version of the NEO PI-R has been used in the Korean population with good reliability and validity [45]. This instrument has a robust factor structure that has been replicated in more than 50 other cultures [16] in addition to that of Korea [45, 46]. It measures 30 facets, six for each of the five major dimensions of personality. Items were answered on a 5-point Likert-type scale with responses ranging from “strongly disagree” to “strongly agree.” There were no missing responses in our dataset, because the subjects could complete the questionnaire if there are no missing data in every page online. In the present study, the Cronbach’s alpha coefficients for neuroticism, extraversion, openness, agreeableness, and conscientiousness were 0.83, 0.82, 0.76, 0.69, and 0.78, respectively. Raw scores were converted to *T*-scores (mean = 50, standard deviation = 1) using Korean combined-sex norms (N = 7,418) to confirm similarities with the Korean normative sample data (PSI Consulting Corp., Seoul, Korea).

### Demographic and behavioral characteristics

Age, marital status, working status, education, caffeine intake, alcohol use, smoking, and physical activity were considered covariates in this analysis. Marital status was categorized as “married” or “single/separated/divorced.” Working status was categorized as “employed” or “unemployed.” Education was categorized as “high school,” “college,” “university,” or “graduated university.” Participants were asked how many alcoholic drinks and caffeinated beverages (coffee, tea, other) they consumed on average per day. Participants were also categorized as “never smokers,” “former smokers,” or “current smokers.” Physical activity was categorized as “low,” “moderate,” or “high” following the categorical scoring of the International Physical Activity Questionnaire (IPAQ) [47].

### Statistical Analysis

All statistical analyses were conducted using SAS version 9.3 for Windows and RStudio version 0.98.983 for Mac. Descriptive statistics were calculated to characterize the sample. Sleep quality was categorized as good (PSQI <6) or poor (PSQI ≥6). The two-sample t-test was used to compare the means of continuous variables of interest for these two groups, while the chi-square test was used to examine differences in categorical variables.

Pearson (for continuous variables) or Spearman (for categorical variables) correlation coefficients (zero-order *r*) were used in preliminary investigations of the relationships among all variables. Sleep quality residuals were normally distributed, and multicollinearity diagnostics were performed on the five personality factors. Next, we performed multivariate linear regression analysis using two models. In Model I, five linear regression analyses were conducted on each personality factor to examine its unique effect on the global PSQI score. In Model II, one multiple linear regression analysis including all five personality factors was conducted, to identify independent predictors of the global PSQI score. The covariates were only used in the regression model if the bivariate correlation indicated they were significantly correlated (p<.05) with the global PSQI score. All statistical tests were two-sided (α = .05).

To further analyze the relationships among the personality traits in the multiple regression models, we examined structure coefficients as well as beta weights (β) and performed commonality analyses. A structure coefficient (*r*_s_) is a correlation between an observed predictor variable and the predicted criterion score (Ŷ) [48]. It is calculated by dividing the Pearson correlation (*r*) between an independent variable and the dependent variable by the multiple correlation (*R*). A squared structure coefficient (*r*_*s*_^2^) reveals how much variance the predictor can explain of the observed *R* ^*2*^ effect (not of the total dependent variable), and therefore provides a sense of how much each predictor contributes to the explanatory power of the entire model [48]. Commonality analysis provides separate measures of unique variance explained for each predictor in addition to measures of shared variance for all combinations of predictors [49].

Next, multivariate logistic regression analysis was conducted to identify independent determinants of poor sleep (global PSQI score ≥6) using two models. In Model I, five different logistic regression analyses were conducted on each personality factor to examine its unique effect on poor sleep. In Model II, one multiple logistic regression analysis including all five personality factors was conducted, to identify independent predictors of poor sleep. All demographic variables were included in both models. In addition, we performed a stepwise discriminant function analysis (DFA) to determine which of five personality factors could best differentiate good sleepers and poor sleepers. The stepwise method of discriminant analyses was preferred over the standard method in order to eliminate discriminating variables that do not add to the power of the discriminant functions.

## Results

### Sample characteristics

Descriptive statistics for the participants are shown in Table 1. Their average age was 33.1±3.9, and roughly 80% of participants were married. The number of employed and unemployed women was roughly equal. Most participants had an educational background of university or higher (71%) and were never smokers (90%). In a univariate analyses, we found no significant associations between demographic and behavioral variables and sleep quality, except for smoking status. The mean sleep quality (PSQI) score was 4.03 (SD = 2.29), and poor sleepers represented 22% of the total. The mean of our subjects was lower than those reported in university students and older women in Caucasian [23, 29, 50]. Overall, the T-scores of personality traits for the entire sample, which never deviate more than 10 points from the standardized men score of 50, indicated that they don’t differ substantially from the normative sample data.

**Table 1:** Descriptive statistics for study participants.

		Sleep quality		
Variable	Overall	Good (PSQI <6)	Poor (PSQI ≥6)	*p*
Age ^a^	33.34 (3.79)	33.40 (3.84)	33.14 (36.2)	0.28
Sex	1,406 (Female)	1,093	313	-
Marital status ^b^				0.33
Married	1,138 (81)	888 (81)	250 (80)	
Single/Separated/Divorced	268 (19)	205 (19)	63 (20)	
Working status ^b^				0.08
Employed	736 (52)	586 (54)	150 (48)	
Unemployed	670 (48)	507 (46)	163 (52)	
Education ^b^				0.77
High school	149 (11)	118 (11)	31 (10)	
College	235 (17)	177 (16)	58 (19)	
University	751 (53)	588 (54)	163 (52)	
≥Graduate school	271 (19)	210 (19)	61 (19)	
Caffeine per Day ^a^	1.22 (1.37)	1.24 (1.38)	1.13 (1.34)	0.22
Alcohol per Day ^a^	2.62 (2.81)	2.61 (2.81)	2.70 (2.85)	0.62
Smoking ^b^				0.01
Never smoker	1,262 (90)	993 (91)	269 (86)	
Former/Current smoker	144 (10)	100 (9)	44 (14)	
Physical Activity ^b^				0.98
Low	813 (58)	632 (58)	181 (58)	
Moderate	486 (34)	377 (34)	109 (35)	
High	107 (8)	84 (8)	23 (7)	
PSQI ^a^				
Subjective sleep quality	1.12 (0.29)	0.95 (0.46)	1.73 (0.58)	<0.001
Sleep latency	0.77 (0.82)	0.56 (0.64)	1.51 (0.93)	<0.001
Sleep duration	0.53 (0.74)	0.34 (0.58)	1.19 (0.86)	<0.001
Habitual sleep efficiency	0.24 (0.62)	0.07 (0.28)	0.85 (0.99)	<0.001
Sleep disturbances range	0.7 (0.48)	0.63 (0.49)	0.95 (0.38)	<0.001
Use of sleeping medication	0.01 (0.11)	0.00 (0.06)	0.03 (0.21)	<0.001
Daytime dysfunction	0.65 (0.68)	0.52 (0.59)	1.12 (0.76)	<0.001
Global score (scale range 0–21)	4.03 (2.29)	3.07 (1.31)	7.39 (1.73)	<0.001
Personality ^a^ (*T*-score, mean = 50, SD = 10)				
Neuroticism	55.18 (9.16)	54.74 (9.12)	56.74 (9.12)	<0.001
Extraversion	44.85 (9.75)	44.93 (9.78)	44.57 (9.67)	0.56
Openness	54.06 (12.52)	53.85 (12.55)	54.81 (12.41)	0.23
Agreeableness	48.41 (11.77)	48.65 (11.72)	47.58 (11.95)	0.16
Conscientiousness	41.98 (8.90)	42.46 (8.88)	40.31 (8.77)	<0.001

*Note*. PSQI: Pittsburgh Sleep Quality Index; SD: Standard Deviation

^a^ Quantitative variable. Mean (standard deviation) and p-value from t-test are shown.

^b^ Nominal scale. Frequency (percentage) and p-value from χ^2^ test are shown.

### Association between personality traits and sleep quality

Table 2 provides the correlation coefficients among all variables. All dimensions except openness were significantly associated with sleep quality. Neuroticism and conscientiousness had a significant correlation with PSQI scores (*r* = 0.170 and -0.104, respectively). Big five personality traits showed non-zero correlations with each other. Among them, neuroticism and conscientiousness had the highest Pearson’s correlation coefficient (*r* = -0.448, *p*<.001). The correlation matrix among the five personality traits indicated that the absolute values of the correlation coefficients were lower than the acceptable cut-off point of 0.8 for inclusion in multiple regression analysis [51]. The correlation matrix between the facets of five personality traits and sleep quality was showed in S1 Table.

**Table 2:** Correlation coefficients for sleep quality, covariates, and personality traits.

Variable	1	2	3	4	5	6	7	8	9	10	11	12	13	14
1. PSQI global score ^a^	1													
2. Age ^a^	-0.075**	1												
3. Marital status ^b^	0.056*	-0.377***	1											
4. Education ^b^	-0.006	-0.055*	0.044*	1										
5. Caffeine ^a^	-0.019	0.111***	0.045	-0.012	1									
6. Alcohol ^a^	0.043	-0.167***	0.200***	-0.109***	0.080**	1								
7. Smoking ^b^	0.064*	-0.028	0.129***	0.010	0.028	0.109***	1							
8. Physical activity ^b^	-0.009	-0.018	0.102***	0.035	0.005	0.043	0.031	1						
9. Working status ^b^	-0.074**	-0.171***	0.274***	0.145***	0.128***	0.169***	0.055*	0.031	1					
10. Neuroticism ^a^	0.170***	-0.084**	0.015	-0.074**	-0.015	-0.014	0.013	-0.084**	-0.089**	1				
11. Extraversion ^a^	-0.063*	-0.058*	-0.002	0.013	0.006	0.120***	0.040	0.104***	0.066*	-0.262***	1			
12. Openness ^a^	0.035	-0.127***	0.138***	0.167***	-0.022	0.069**	0.072*	0.115***	0.043	-0.085**	0.399***	1		
13. Agreeableness ^a^	-0.095***	0.171***	-0.116***	-0.009	-0.061*	-0.107***	-0.102***	-0.017	-0.088**	-0.231***	0.023	-0.001	1	
14. Conscientiousness ^a^	-0.104***	0.054*	0.014	0.150***	0.009	-0.046	-0.057*	0.144***	0.109***	-0.448***	0.204***	0.201***	0.065*	1

N = 1,406

^a^ Pearson’s correlation coefficients

^b^ Spearman correlation coefficients

*p<.05

**p<.01

***p<.001

Although the absolute values of the correlation coefficients between the five dimensions of personality were lower than the acceptable cut-off point of 0.8, we investigated potential multicollinearity among the variables by calculating variance inflation factors (VIFs) and tolerance values for the multiple regression analysis. VIF scores and tolerance values ranged from 1.120 to 1.400 and from 0.715 to 0.893, respectively, indicating that multicollinearity was not a problem in our model.

Associations between personality traits and sleep quality (continuous PSQI global scores), controlling for demographic and behavioral variables, are shown in Table 3. The results of a series of linear regression analyses for each individual personality domain (Model I) showed that all dimensions except openness were significantly associated with sleep quality. With a standardized regression (β) weight of 0.157 (*P*<.001), neuroticism showed the strongest positive association with PSQI scores. Extraversion, agreeableness, and conscientiousness showed significant negative associations with PSQI scores. In a multiple linear regression model (Model II), the combination of age, marital status, smoking, and working status explained 1.9% of the variance on sleep quality. The five personality dimensions explained an additional 3.1% of variance in sleep quality. The multiple regression equation including all five personality domains was significant at the 0.01 level (*F* = 8.08, *p*<.001, *R* ^2^ = 0.050, Adjusted *R* ^2^ = 0.043). Of the five personality dimensions, neuroticism was found to contribute most significantly to the prediction of sleep quality (β = -0.123, *p*<.001). Although extraversion, agreeableness, and conscientiousness were significantly correlated with sleep quality in zero-order bivariate analyses, in combination with the other personality domains they were not significant predictors. In contrast, openness was shown significant association with sleep quality in the multiple linear regressions, although it was not correlated with sleep quality in zero-order bivariate analyses.

**Table 3:** Linear regression analysis investigating the association between personality traits and sleep quality, as measured by PSQI global score.

Factors	Model I ^a^	Model II ^b^				
	β ^c^	β ^c^	Structure Coefficient (*r*_*s*_)	*r*_*s*_^2^	Unique ^d^	Common ^e^
Neuroticism (N)	0.157***	0.123***	0.764	0.584	0.011	0.018
Extraversion (E)	-0.065*	-0.047	-0.285	0.081	0.002	0.002
Openness (O)	0.021	0.061*	0.156	0.024	0.003	-0.002
Agreeableness (A)	-0.084**	-0.053	-0.428	0.183	0.003	0.007
Conscientiousness (C)	-0.087***	-0.033	-0.467	0.218	0.001	0.010

N = 1,406

*Note*. All models were adjusted for age, marital status, smoking, and working status.

^a^ Model I: linear regression model including a single domain of personality as an independent variable (R2 = 0.043, 0.023, 0.020, 0.026, and 0.027; adjusted R^2^ = 0.040, 0.020, 0.016, 0.022, and 0.023, *F* = 12.67, 6.70, 5.61, 7.45, and 7.68; *P*<.001, *<*.001, <0.001, *<*.001, and <0.001 in N, E, O, A, and C, respectively).

^b^ Model II: multiple linear regression model including all five domains of personality as independent variables (R^2^ = 0.0524, adjusted R^2^ = 0.041, *F* = 4.80, *P*<.001).

^c^ β: standardized coefficient in linear regression analyses.

^d^ Unique = proportion of criterion variance explained uniquely by the predictor.

^e^ Common = proportion of criterion variance explained by the predictor that is also explained by one or more other predictors. Unique + Common = *r* ^2^, *r* = zero-order correlation coefficient

*p <.05

**p <.01

***p<.001

The associations between the facets of five personality traits and sleep quality were showed in S1 Table. At facet level, anxiety (N1) depression (N3), and impulsiveness (N5), facets of neuroticism, were positively associated with PSQI global scores. Gregariousness (E2), activity (E4), and positive emotions (E6) of extraversion, feeling (O3) and values (O6) of openness, trust (A1) and straightforwardness (A2) of agreeableness, and self-discipline (C5) of conscientiousness were negatively associated with PSQI global scores.

We considered structure coefficients (*r*_*s*_) to interpret the multiple linear regression as well as beta weights. If two or more predictors explain some of the same part of the criterion, the sum of squared structure coefficients for all predictors will be greater than 1.00 [52]. In our model, the sum of five dimensions of personality was 1.090, suggesting a small amount of multicollinearity was present. After computing the structure coefficients for this multiple regression model, neuroticism was still the most noteworthy predictor of sleep quality (*r*_*s*_ = 0.764) and could account for 58.4% (*r*_*s*_^2^ = 0.584, partial *R* ^2^ = 0.029) of the explained variance (*R* ^2^ = 0.050). The rank of structure coefficient for conscientiousness was second (*r*_*s*_ = -0.467, *r*_*s*_^2^ = 0.218) among the five personality factors, not fifth as the beta weights suggested. Openness showed the smallest structure coefficient and could account for 2% of the obtained effect by itself (*r*_*s*_ = 0.156, *r*_*s*_^2^ = 0.024), although the beta weight was significant and the rank of that was second. Yet openness has insignificant zero-order correlation and small structure coefficients with sleep quality but a sizeable non-zero beta weight. In this case openness is serving as a suppressor variable and helping the other predictor variables do a better job of predicting the sleep quality even though openness itself is unrelated to the outcome. This affect *R* ^2^ by allowing the beta weights for other predictors to deviate further from zero than the boundary of their respective zero-order correlations with sleep quality, thus making some of the other product terms within the equation larger, and thus making *R* ^2^ larger [27]. Inspection of the structure coefficients suggests that neuroticism was a very strong indicator of PSQI scores, conscientiousness was a moderate indicator of PSQI scores, and openness was a suppressor variable in the multiple regression models.

We considered commonality coefficients to find why the conscientiousness vanished the significance of a beta weight in multiple linear regression models although its contribution for PSQI scores was identified by correlation coefficients and structure coefficients. Commonality analysis decomposes the squared multiple correlation (*R* ^2^) to determine both the unique and common explained variance of the sleep quality by the predictor (Table 3 and S2 Table). In total, the five personality domains individually accounted for 38% (sum of the unique/*R* ^2^ = 0.019/0.050) of the regression effect. The remaining 62% was due to variance in PSQI global scores that the predictors shared. The unique coefficient for neuroticism (0.011) indicated that neuroticism uniquely explained 1.1% of the variance in PSQI scores. This amount of variance is more than any other partition, representing 22.03% of the *R* ^2^ effect (0.050). The unique coefficient for conscientiousness (0.001) was the smallest of the unique effects and indicated that the regression model only improved slightly with the addition of conscientiousness. The common effects represented the proportion of PSQI scores variance that could be jointly explained by two or more predictors together. Neuroticism and conscientiousness together explained 0.5% of the outcome, which represented 10.92% of the total effect size (S2 Table). The common effects also showed that openness served as a suppressor variable, yielding a unique effect greater than its total contribution to the regression effect and negative commonality coefficients [25]. Overall, commonality analysis could help to quantify the location and amount of variance explained by suppression and multicollinearity. We also investigated the potential interaction between neuroticism and conscientiousness in predicting sleep quality. In a regression analysis including neuroticism, conscientiousness and their interaction, the interaction term was not significant (β = -0.118, *p* = 0.380).

Additionally, S3 Table was shown the associations between five personality traits and seven components of sleep quality as measured by PSQI. The subjective sleep quality scores were associated with extraversion and openness. The sleep latency scores were showed the positive association with neuroticism but the negative association with extraversion. The sleep duration scores and use of sleeping medication scores were not significantly associated with any personality trait. The habitual sleep efficiency scores were negatively associated with conscientiousness. The daytime dysfunction scores were shown the positive association with neuroticism.

### Personality factors and facets predict poor sleepers

Results from the t-test indicated significant personality differences between sleep quality groups (Table 1). Neuroticism score was higher in poor sleepers than good sleeper (t = -3.42, p<0.001), while conscientiousness was lower in poor sleepers than good sleepers (t = 3.79, p<0.001). The other personality domains showed no difference between groups.

As noted earlier, a logistic regression analysis was used to examine whether personality traits could predict poor quality status identified by PSQI scores (global PSQI ≥6) (Table 4). In the logistic regression models that considered the personality traits individually (Model I), both neuroticism and conscientiousness were significant, separate predictors of poor sleep quality status. The significant association between neuroticism and poor sleep in Model I vanished when investigated in a multiple regression model adding the other personality domains (Model II). In contrast to the results of the linear regression, conscientiousness was the most noteworthy predictor of sleep quality in logistic regression models (Model II). Individuals high in conscientiousness were least likely to have poor sleep quality, which is consistent with an OR of 0.813, with conscientiousness being protective against poor sleep quality, and specifies that the logistic regressions are predicting poor sleep quality status rather than good sleep quality status. The facets of neuroticism and conscientiousness were analyzed using the same models (Model I and II). Among the facets of neuroticism, depression (N3) were the strongest predictors of poor sleep quality status (OR = 1.175, 95% CI = 1.004 to 1.375). The score of deliberation (C6) was significantly lower in poor sleepers than in good sleepers (OR = 0.878, 95% CI = 0.781 to 0.988).

**Table 4:** Logistic regression models predicting poor sleep quality based on personality characteristics.

	Poor sleepers, n = 313 (reference: good sleepers, n = 1,093)	
	OR ^a^ (95% CI)	
Factors	Model I ^b^	Model II ^c^
Neuroticism (N)	1.246 (1.084, 1.433)**	1.137 (0.966, 1.338)
Extraversion (E)	0.960 (0.843, 1.093)	0.982 (0.848, 1.138)
Openness to Experience (O)	1.050 (0.947, 1.163)	1.101 (0.983, 1.233)
Agreeableness (A)	0.939 (0.842, 1.047)	0.966 (0.863, 1.083)
Conscientiousness (C)	0.784 (0.680, 0.904)***	0.813 (0.692, 0.956)*
Facets ^d^		
N1: Anxiety	1.150 (1.015, 1.303)*	1.075 (0.923, 1.253)
N2: Angry Hostility	1.127 (1.007, 1.261)*	1.012 (0.885, 1.157)
N3: Depression	1.211 (1.076, 1.381)**	1.175 (1.004, 1.375)*
N4: Self-consciousness	1.043 (0.936, 1.161)	0.914 (0.800, 1.044)
N5: Impulsiveness	1.178(1.047, 1.326)**	1.137 (0.999, 1.294)
N6: Vulnerability	1.127 (1.006, 1.263)*	1.012 (0.875, 1.171)
C1: Competence	0.896 (0.805, 0.999)*	0.945 (0.839, 1.065)
C2: Order	0.949 (0.832, 1.082)	1.110 (0.947, 1.302)
C3: Dutifulness	0.879 (0.782, 0.988)*	0.961 (0.837, 1.104)
C4: Achievement Striving	0.873 (0.784, 0.971)*	0.943 (0.821, 1.084)
C5: Self-Discipline	0.844 (0.749, 0.952)**	0.905 (0.782, 1.047)
C6: Deliberation	0.856 (0.770, 0.951)**	0.878 (0.781, 0.988)*

N = 1,406

*Note*. CI: Wald Confidence Interval

^a^ Odds Ratios (ORs) per 10 *T*-score increase in a given personality trait, controlling for age, marital status, working status, education, caffeine intake, alcohol use, smoking status, and physical activity.

^b^ Model I: logistic regression model including a single domain or facet of personality as an independent variable.

^c^ Model II: multiple logistic regression model including all five domains of personality as independent variables. At a facet level, six facets of each domain were included in the model.

^d^ Logistic regression analyses of the facet level were performed in neuroticism and conscientiousness.

*p<.05

**p<.01

***p<.001

Because of slightly inter-correlation among some of the five personality domains, a stepwise DFA was computed with covariates to evaluate which measures uniquely maximized the separation between good sleepers and poor sleepers (Table 5). This analysis also allowed us to determine a more parsimonious set of personality traits. The stepwise DFA indicated conscientiousness and neuroticism were statistically significant and independent contributors to group separation. The classification accuracy of the DFA model was 60.4% for good sleepers and 53.4% for poor sleepers. At facet levels, depression (N3), impulsiveness (N5), self-discipline (C5), and deliberation (C6) were statistically significant and independent contributors to group separation. The classification accuracies for the specific facets of neuroticism and conscientiousness were 59.6% and 60.8% for good sleepers, respectively, and 54.3% and 49.5% for poor sleepers, respectively.

**Table 5:** Discriminant function analysis (DFA) by the stepwise method for the separation between good sleepers and poor sleepers.

Variable	Standardized coefficients	Wilk’s λ	*F*	*p*	DFA entry order
Factors					
Neuroticism	0.452	0.983	7.916	<0.0001	2
Conscientiousness	-0.553	0.990	14.396	<0.0001	1
Smoking (covariate)	0.488	0.986	9.933	<0.0001	3
Facets: Neuroticism					
N3: Depression	0.605	0.992	11.572	0.001	1
N5: Impulsiveness	0.470	0.984	7.554	<0.0001	2
Smoking (covariate)	0.494	0.987	8.991	<0.0001	3
Facets: Conscientiousness					
C5: Self-Discipline	0.550	0.993	9.519	0.002	1
C6: Deliberation	0.497	0.986	6.753	<0.0001	2
Smoking (covariate)	-0.521	0.989	7.797	<0.001	3

N = 1,406

### Supplementary Analyses

To determine the robustness of our findings, we re-ran analyses using PCA-derived personality factors, as described by Duggan et al. [23]. PCA-derived personality factors obtained using the orthogonal varimax rotation. Thus, PCA removed the substantial multicollinearity between the personality traits. The results were consistent with the findings of bivariate analyses and Model I of logistic analyses (S4 Table). In the simultaneous multiple linear regression model, PCA-derived neuroticism showed a positive association with PSQI global scores (β = 0.136, *p*<.001), while PCA-derived extraversion, agreeableness, and conscientiousness were negatively associated with the scores (extraversion: β = -0.052, *p*<.05, agreeableness: β = -0.069, *p*<.01, and conscientiousness: β = -0.059, *p*<.05). In logistic regression models, PCA-derived conscientiousness was the strongest predictor of sleep quality (OR = 0.822, 95% CI = 0.724 to 0.933) while PCA-derived neuroticism was the second-best predictor (OR = 1.171, 95% CI = 1.031 to 1.330). At the facet level, PCA-derived depression (N3), impulsiveness (N5), self-discipline (C5), and deliberation (C6) showed significant associations with sleep quality.

## Discussion

The main findings of this study were that neuroticism and conscientiousness were associated with PSQI global scores, even after controlling for demographic and behavioral characteristics. Among young women not reporting clinically meaningful depression symptoms, poor sleepers scored significantly lower than good sleepers on conscientiousness. We examined not only the factors of personality traits, but also facet-level analysis, which permits the examination of multifaceted constructs, which could be relevant to understanding the relationship between personality and sleep quality. This study examined how both unique and common effects of each personality trait explain variance in sleep quality. In particular, the current study provided a rich interpretation of our sleep quality regression models, by identifying personality trait suppressor effects and patterns of shared variance that would have gone undiscovered through reliance on beta weights alone. In Table 3, we simultaneously considered beta weights, structure coefficients, unique effects, and common effects when evaluating the importance of PSQI global score predictors in the multiple linear regression models. The each coefficient has distinct advantages and limitations, and that multiple statistical measures complement each other in the perspective they provide regarding regression findings [53]. We found the best predictors of sleep quality were different domains of five personality traits in accordance with the approach for the PSQI global score, either continuous variables or discrete variables.

In our multiple linear regression models, we found that neuroticism was the best contributor to PSQI global scores, though all dimensions except openness were significantly associated with PSQI global scores in the bivariate analyses. Conscientiousness was highly correlated with sleep quality in the zero-order bivariate linear relationship, but it was not significant in a multiple linear regression model. Moreover, conscientiousness did not moderate the association between neuroticism and sleep quality. These results were consistent with prior findings [28]. Many researchers have recommended that both beta weights and structure coefficients be considered when interpreting the results of regression models containing correlated predictor variables [25, 27]. Interpreting both beta weights and structure coefficients could help to not only find existence of a small amount of multicollinearity, but also identify the best predictor and suppressor for sleep quality in the multiple linear regressions. We found a reason why conscientiousness was not a significant predictor in multiple regression models. In the commonality analysis, conscientiousness explained more shared than unique variance in PSQI global scores. Williams et al. also examined the unique and interactive effects of neuroticism and conscientiousness for sleep quality using moderation model of the regression analysis, but they interpreted only beta weights in the regression model [28]. We quantified the amount of variance explained by multicollinearity using the commonality analysis. Another interesting finding was openness’s role as a suppressor in predicting PSQI global scores. It was confirmed in both structure coefficients and commonality coefficients.

In the logistic analyses, conscientiousness remained a significant predictor of poor sleep quality status even after inclusion of several lifestyle factors in our models. Several demographic risk factors for poor sleep, such as sex [50], being elderly [50], marriage status [54], education [55], and income[56], have been reported. According to the results of the multiple linear and logistic regressions, we suggest that neuroticism might be the best predictor for sleep quality using PSQI global scores as continuous variables, while conscientiousness might be the best predictor at risk for poor sleep quality status using PSQI global scores as discrete variables, even though neuroticism was the second-best predictor in DFA and PCA.

Our findings replicate and extend previous research on the association between personality traits and sleep quality [28, 29]. At the seven component of sleep quality, neuroticism was associated with relatively subjective component such as sleep disturbances and daytime dysfunction, while it was not related to more objective aspects such as sleep duration. Conscientiousness was the only personality factor associated with the habitual sleep efficiency. The more objective component, such as sleep duration and use of sleeping medication were not shown association with any personality factor. Perhaps people low in conscientiousness and high in neuroticism have poor sleep quality because both factors are associated with difficulty regulating emotions (e.g., anxiety, depression) and behavior (e.g., sleep hygiene) [23]. The propensity for highly neurotic individuals to be sensitive to signs of threat and dysfunction may place them at greater risk for dysfunction in response to perceived sleep disruption [28]. Individuals who score low in conscientiousness tend to engage in risky behaviors that contribute to poor physical health [18].

To the best of our knowledge, this is also the sizeable study to evaluate the effects of personality on sleep quality in Asians. Despite our study’s contributions, it does have some limitations. First, because this study was based on cross-sectional data, we could not control for the confounding influence of time-invariant common causes or infer causal relationships between variables. In addition, this study was confined to women.

Identifying the role of personality traits in sleep quality not only provides insight into the etiology of poor sleep but can suggest points of intervention and help tailor prevention and treatment strategies to individual poor sleepers. For example, when faced with a poor sleeper high in conscientiousness, a physician’s or nurse’s advice to change his or her sleep hygiene or control bed/wake time would likely be met by a high degree of self-directed effort on the part of the patient. However, for poor sleepers low in conscientiousness, this advice may need to be accompanied by short-term incentives, regular monitoring, and behavior modification reminders, either by a healthcare provider or some other expert [57]. In addition, certain personality styles, such as those who are high in neuroticism and low in conscientiousness, could be flagged as being at much higher risk for sleep quality. Such reports could be developed with the help of physician and epidemiologists [57]. A few studies have reported that interventions such as cognitive behavioral therapy (CBT), interpersonal psychotherapy, or generic therapy were effective in the reduction of neuroticism levels [58, 59], anxiety, and depression [60] or in the increasing conscientiousness [59, 61]. Thus, knowledge of patients’ personality profiles could aid in differential treatment planning [62].

In conclusion, this study demonstrated that neuroticism is the strongest personality factor influencing PSQI global scores and conscientiousness is the best predictor of poor quality status in young Korean women. Personality may account for a relatively small but significant amount (~4%) of the variance in sleep quality. However, given the prevalence of sleep disturbance worldwide, even small improvements could have considerable public health impact. Early intervention and prevention efforts targeting sleep quality are important for improving the health of young women.

## Supporting Information

S1 Table Correlations between PSQI global score and facets of five personality domains. (DOC)

S2 Table Commonality coefficients. (XLS)

S3 Table Associations between personality traits and the basic components of sleep quality as measured by PSQI. (DOC)

S4 Table Multiple regressions of sleep quality and PCA-derived personality components. (DOC)
