# Supplementary material for: Association between Personality Traits and Sleep Quality in Young Korean Women
Source: PLoS One. 2015 Jun 1;10(6):e0129599. doi: 10.1371/journal.pone.0129599 (PMC4452145; doi:10.1371/journal.pone.0129599)
Supplement: S1 Table — (DOC) [file pone.0129599.s001.doc]

**Table S1. Correlations between** PSQI global score and facets of five personality domains

| Facets |  | Pearson’s correlation coefficients | *p* |  | Standardized coefficients ^a^ | *p* |
| --- | --- | --- | --- | --- | --- | --- |
| N1: Anxiety |  | 0.131^***^ | < 0.001 |  | 0.071^*^ | 0.026 |
| N2: Angry Hostility |  | 0.110^***^ | < 0.001 |  | 0.015 | 0.630 |
| N3: Depression |  | 0.162^***^ | < 0.001 |  | 0.107^**^ | 0.002 |
| N4: Self-consciousness |  | 0.076^**^ | 0.005 |  | -0.037 | 0.255 |
| N5: Impulsiveness |  | 0.116^***^ | < 0.001 |  | 0.062^*^ | 0.031 |
| N6: Vulnerability |  | 0.117^***^ | < 0.001 |  | 0.011 | 0.742 |
| E1: Warmth |  | -0.014 | 0.612 |  | 0.062 | 0.070 |
| E2: Gregariousness |  | -0.066^*^ | 0.014 |  | -0.081^*^ | 0.011 |
| E3: Assertiveness |  | -0.033 | 0.213 |  | 0.006 | 0.854 |
| E4: Activity |  | -0.091^**^ | 0.001 |  | -0.072^*^ | 0.022 |
| E5: Excitement Seeking |  | 0.009 | 0.748 |  | 0.048 | 0.113 |
| E6: Positive Emotions |  | -0.080^**^ | 0.003 |  | -0.074^*^ | 0.015 |
| O1: Fantasy |  | 0.033 | 0.214 |  | -0.003 | 0.920 |
| O2: Aesthetics |  | 0.055^*^ | 0.039 |  | 0.043 | 0.173 |
| O3: Feelings |  | 0.076^**^ | 0.004 |  | 0.069^*^ | 0.022 |
| O4: Actions |  | 0.021 | 0.425 |  | -0.007 | 0.803 |
| O5: Ideas |  | 0.005 | 0.858 |  | -0.018 | 0.546 |
| O6: Values |  | -0.072^**^ | 0.007 |  | -0.085^**^ | 0.002 |
| A1: Trust |  | -0.105^***^ | < 0.001 |  | -0.083^**^ | 0.004 |
| A2: Straightforwardness |  | -0.088^**^ | 0.001 |  | -0.061^*^ | 0.033 |
| A3: Altruism |  | -0.037 | 0.167 |  | 0.008 | 0.787 |
| A4: Compliance |  | -0.060^*^ | 0.024 |  | -0.021 | 0.468 |
| A5: Modesty |  | -0.022 | 0.406 |  | 0.020 | 0.493 |
| A6: Tender-Mindedness |  | -0.018 | 0.500 |  | -0.002 | 0.937 |
| C1: Competence |  | -0.061^*^ | 0.023 |  | -0.012 | 0.668 |
| C2: Order |  | -0.022 | 0.415 |  | 0.053 | 0.093 |
| C3: Dutifulness |  | -0.078^**^ | 0.003 |  | -0.016 | 0.622 |
| C4: Achievement Striving |  | -0.088 | 0.111 |  | -0.043 | 0.213 |
| C5: Self-Discipline |  | -0.119^***^ | <0.001 |  | -0.091^**^ | 0.005 |
| C6: Deliberation |  | -0.040 | 0.134 |  | -0.014 | 0.630 |

^a^ Multiple linear regression model included six facets of each personality domain as independent variables. All analyses were adjusted for age, marital status, working status, and smoking status.

^*^*p*<.05, ^**^*p*<.01, ^***^*p*<.001
