# Supplementary material for: Association between Personality Traits and Sleep Quality in Young Korean Women
Source: PLoS One. 2015 Jun 1;10(6):e0129599. doi: 10.1371/journal.pone.0129599 (PMC4452145; doi:10.1371/journal.pone.0129599)
Supplement: S3 Table — (DOC) [file pone.0129599.s003.doc]

**Table S3. Associations between personality traits and the basic components of sleep quality as measured by PSQI**

| Variable | Subjective sleep quality | Sleep latency | Sleep duration | Habitual sleep efficiency | Sleep disturbances range | Use of sleeping medication | Daytime dysfunction |
| --- | --- | --- | --- | --- | --- | --- | --- |
| Correlation coefficients (*r*)^a^ |  |  |  |  |  |  |  |
| Neuroticism | 0.105^***^ | 0.143^***^ | 0.027 | 0.039 | 0.100^***^ | 0.029 | 0.168^***^ |
| Extraversion | -0.064^*^ | -0.103^***^ | 0.008 | -0.007 | 0.009 | 0.017 | -0.045 |
| Openness | 0.018 | -0.017 | 0.051 | 0.009 | 0.015 | -0.022 | 0.050 |
| Agreeableness | -0.051 | -0.025 | -0.080^**^ | -0.037 | -0.071^**^ | 0.008 | -0.074^**^ |
| Conscientiousness | -0.102^***^ | -0.082^**^ | 0.011 | -0.082^**^ | -0.031 | -0.034 | -0.071^**^ |
| Standardized coefficients (β)^b^ |  |  |  |  |  |  |  |
| Neuroticism | 0.046 | 0.112^***^ | 0.025 | -0.015 | 0.102^**^ | 0.031 | 0.159^***^ |
| Extraversion | -0.066^*^ | -0.079^*^ | -0.001 | -0.009 | 0.020 | 0.044 | -0.032 |
| Openness | 0.062^*^ | 0.025 | 0.035 | 0.030 | 0.001 | -0.032 | 0.047 |
| Agreeableness | -0.039 | 0.005 | -0.053 | -0.043 | -0.048 | 0.009 | -0.013 |
| Conscientiousness | -0.053 | 0.004 | 0.008 | -0.071^*^ | 0.024 | -0.033 | -0.022 |
| *R*^2^ | 0.050 | 0.056 | 0.036 | 0.037 | 0.038 | 0.009 | 0.062 |
| Adusted *R*^2^ | 0.039 | 0.045 | 0.025 | 0.026 | 0.027 | -0.003 | 0.051 |
| *F* | 4.53^***^ | 5.13^***^ | 3.28^***^ | 3.35^***^ | 3.40^***^ | 0.75 | 5.72^***^ |

N=1,406

^a^ Pearson’s correlation coefficients

^b^ The multiple linear regression models included all five domains of personality as independent variables. All analyses were adjusted for age, marital status, working status, education, caffeine intake, alcohol use, smoking status, and physical activity.

^*^ p <.05, ^**^p <.01. ^***^p <.001

PSQI: Pittsburgh Sleep Quality Index
