# Supplementary material for: Association between Personality Traits and Sleep Quality in Young Korean Women
Source: PLoS One. 2015 Jun 1;10(6):e0129599. doi: 10.1371/journal.pone.0129599 (PMC4452145; doi:10.1371/journal.pone.0129599)
Supplement: S4 Table — (DOC) [file pone.0129599.s004.doc]

**Table S4. Multiple regressions of sleep quality and PCA-derived personality components**

| Variables | Linear model ^a^ | Logistic model ^c^ |
| --- | --- | --- |
|  | β ^b^ | OR (95% CI) ^d^ |
| Age | -0.051 | 0.990 (0.954, 1.028) |
| Marital status | 0.018 | 1.028 (0.710, 1.489) |
| Smoking status | 0.063^*^ | 1.533 (1.037, 2.266)^*^ |
| Working status | -0.079^**^ | 0.802 (0.613, 1.050) |
| Factors |  |  |
| Neuroticism | 0.136^***^ | 1.171 (1.031, 1.330)^*^ |
| Extraversion | -0.052^*^ | 0.977 (0.860, 1.110) |
| Openness | 0.044 | 1.099 (0.966, 1.250) |
| Agreeableness | -0.069^**^ | 0.943 (0.828, 1.074) |
| Conscientiousness | -0.059^*^ | 0.822 (0.724, 0.933)^**^ |
| Facets ^e^ |  |  |
| N1: Anxiety |  | 0.908 (0.798, 1.033) |
| N2: Angry Hostility |  | 0.934 (0.823, 1.060) |
| N3: Depression |  | 0.846 (0.745, 0.960)^**^ |
| N4: Self-consciousness |  | 1.037 (0.913, 1.178) |
| N5: Impulsiveness |  | 0.861 (0.758, 0.978)^*^ |
| N6: Vulnerability |  | 0.940 (0.827, 1.068) |
| C1: Competence |  | 0.910 (0.802, 1.033) |
| C2: Order |  | 1.034 (0.910, 1.174) |
| C3: Dutifulness |  | 0.923 (0.812, 1.048) |
| C4: Achievement Striving |  | 0.906 (0.797, 1.029) |
| C5: Self-Discipline |  | 0.878 (0.775, 0.996)^*^ |
| C6: Deliberation |  | 0.852 (0.751, 0.967)^*^ |

*Note*. CI: Wald Confidence Interval

^a^ Multiple linear regression model including all five domains of PCA-derived personality as independent variables (*R*^2^=0.050, adjusted *R*^2^=0.043 *F*=8.08, *p*<.001 )

^b^ β: standardized coefficient in linear regression analyses.

^c^ Multiple logistic regression model including all five domains of personality as independent variables. At a facet level, six facets of each domain were included in the model.

^d^ Odds Ratios (ORs) per 1 unit of PCA-derived personality increase, controlling for age, marital status, smoking status, and working status. Reference is good sleepers.

^e^ Logistic regression analyses of the facet level were performed in neuroticism and conscientiousness.

^*^*p*<.05, ^**^*p*<.01, ^***^*p*<.001
